# Diffuse large B cell lymphoma involving Meckel’s cave masquerading as biopsy-negative giant cell arteritis: a case report

**DOI:** 10.1186/s13256-020-02379-9

**Published:** 2020-05-10

**Authors:** Matthew J. Samec, Andres G. Madrigal, Charlotte H. Rydberg, Matthew J. Koster

**Affiliations:** 1 Department of Internal Medicine, Mayo Clinic College of Medicine and Science, Rochester, MN 55905 USA; 2 Department of Hematopathology, Mayo Clinic College of Medicine and Science, Rochester, MN 55905 USA; 3 Department of Radiology, Mayo Clinic College of Medicine and Science, Rochester, MN 55905 USA; 4 Division of Rheumatology, Mayo Clinic College of Medicine and Science, Rochester, MN 55905 USA

**Keywords:** Lymphoma, Metastatic, Trigeminal nerve, Giant cell arteritis

## Abstract

**Background:**

Given the absence of consensus diagnostic criteria for giant cell arteritis, clinicians may encounter difficulty with identification of new-onset headache in patients older than age 50 years presenting with visual changes and elevated inflammatory markers, particularly if temporal artery biopsies are performed and negative.

**Case presentation:**

We present a case of a 57-year-old white man with headache, diplopia, and jaw paresthesia initially diagnosed and managed as steroid-refractory biopsy-negative giant cell arteritis. Further investigation disclosed evidence of soft tissue infiltration into Meckel’s (trigeminal) cave bilaterally. Positron emission tomography suggested the presence of a lymphoproliferative disorder. Histology confirmed the diagnosis of diffuse large B cell lymphoma.

**Conclusions:**

Metastatic involvement in Meckel’s cave in diffuse large B cell lymphoma is extremely rare and presents a diagnostic challenge. Patients with suspicion of giant cell arteritis should undergo advanced imaging, particularly those with negative biopsy, atypical features, or lack of response to standard therapy, in order to assess for the presence of large-vessel vasculitis or other mimicking pathologies.

## Background

The diagnosis of giant cell arteritis (GCA) is based on clinical assessment of signs, symptoms, and laboratory features suggestive of the disease. Although non-invasive arterial imaging has shown increasing promise, the gold standard for diagnosis is confirmation through positive temporal artery biopsy (TAB) [1, 2]. Due to the lack of validated and approved diagnostic criteria for GCA, clinicians may inaccurately use criteria intended for research classification to diagnose this condition [3]. Here we describe a case of elevated inflammatory markers, frontal headache, and visions changes that was initially diagnosed as biopsy-negative GCA for which additional evaluation revealed a rare mimicking condition.

## Case description

A 57-year-old white man presented with a 3-month history of new-onset, severe, bi-frontal headaches and a 13.6 kg (30-lb) weight loss. Local emergency room evaluation revealed a negative computed tomography (CT) scan of his head. Laboratory findings included an elevated erythrocyte sedimentation rate (ESR) of 80 mm/hour and C-reactive protein (CRP) of 55.3 mg/L. He was discharged with pain control.

During follow-up with his local primary provider, his headache persisted and bilateral jaw pain and left facial numbness had developed. He was started on prednisone 60 mg/day for presumed GCA with partial improvement in his headache. Bilateral temporal artery biopsies were performed 3 days later and were negative. Prednisone was subsequently discontinued. Two weeks later his headache progressed and right eye horizontal diplopia and perioral numbness developed. Magnetic resonance imaging (MRI) of his brain was performed and interpreted as normal. He was admitted for pulse-dose steroids (1000 mg daily for 3 days) which led to resolution of visual symptoms and was discharged on prednisone 60 mg/day. Tapering below 50 mg/day was unsuccessful due to rising inflammatory markers, symptom progression, and return of diplopia. Severe left hip pain developed for which plain radiographs were obtained but negative for fracture or avascular necrosis.

On referral to our institution he continued to have ongoing headache and left jaw numbness despite 6 weeks of high-dose glucocorticoids (80 mg/day). Left hip pain had worsened to the point of wheelchair dependency. His past medical history was remarkable for atrial fibrillation for which he was receiving warfarin 2 mg/day and diltiazem 240 mg/day. Social history was notable for lack of tobacco or alcohol use and absence of known environmental exposures during his employment as an office manager. Aside from prostate cancer in his father, our patient’s family history was negative for other pertinent diagnoses of additional malignancies or autoimmune conditions. Laboratory evaluation noted an ESR 72 mm/hour, CRP 53 mg/L, hemoglobin 11.1 g/dL, leukocytes 5.2 × 10^9^/L and platelets 110 × 10^9^/L, international normalized ratio (INR) 2.3, creatinine 1.04 mg/dL, calcium 11.6 mg/dL, alanine aminotransferase 106 U/L, aspartate aminotransferase 64 U/L, alkaline phosphatase 360 U/L, total bilirubin 1.2 mg/dL, total protein 5.1 g/dL, and albumin 3.4 g/dL. Previously obtained autoimmune serologies were negative for antinuclear and extractable nuclear antigens as well as rheumatoid factor, anti-cyclic citrullinated peptide, myeloperoxidase, and proteinase-3 antibodies.

His vital signs demonstrated a heart rate of 95 beats per minute, blood pressure of 94/44 mm Hg, respiratory rate of 12 per minute, oxygenation saturation of 96%, and weight of 144 kg with body mass index (BMI) of 48.6 kg/m^2^. An examination was notable for a Cushingoid appearance and morbid obesity. Evaluation of adenopathy and splenomegaly was limited due to body habitus. Pertinent cardiovascular findings included irregularly irregular pulse without murmurs. Upper and lower arterial pulses were normal and symmetric. Common superficial temporal artery palpation was without tenderness or nodularity. Trace pitting edema was noted to the ankle bilaterally. Breath sounds were equal and symmetric without wheezing or rhonchi. Aside from scattered bruising at site of venipuncture, no cutaneous abnormalities were noted. A neurologic examination showed normal speech without language deficits. Visual acuity was 20/20 bilaterally. Cranial nerves 2–12 were assessed and normal except for mild esotropia of his right eye and hypoesthesia to light touch and pinprick over the left mandibular region of the left trigeminal nerve. The remaining dermatomes evaluated showed normal sensation. Reflexes were normal and symmetric throughout and toes were down-going bilaterally. No ataxia was observed. A musculoskeletal examination demonstrated normal range of motion of upper extremities without deficit. Marked pain was noted on passive and active range of motion of his left hip.

**Fig. 1:**
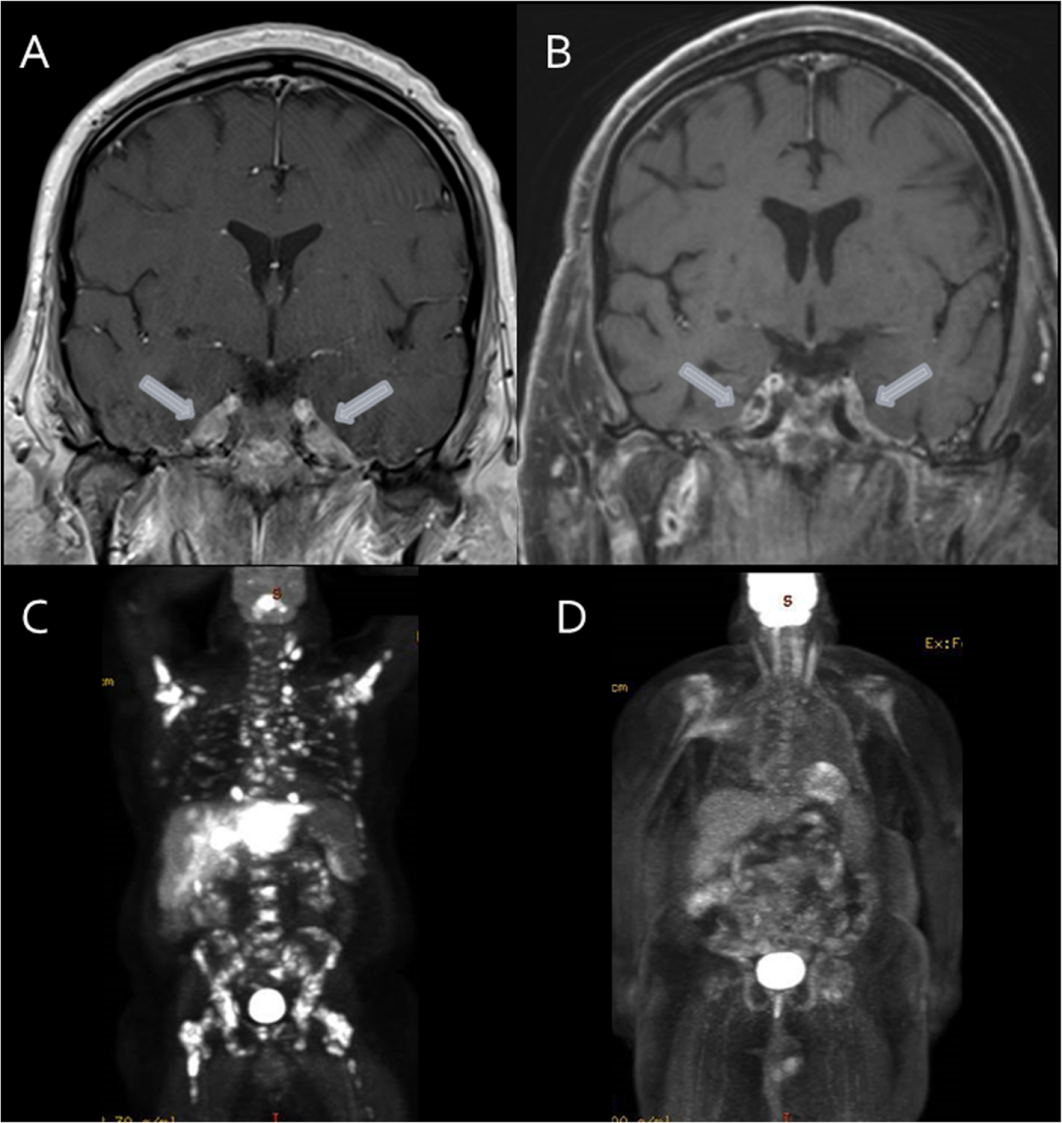
Diffuse large B cell lymphoma involvement on magnetic resonance imaging and positron emission tomography-computed tomography. Magnetic resonance images of brain show: **a** abnormal enhancement of Meckel’s cave bilaterally with extension through the foramen ovale (arrows) before chemotherapy and **b** decreased soft tissue in Meckel’s cave bilaterally (arrows) after chemotherapy. Positron emission tomography-computed tomography scans show: **c** extensive hypermetabolic lesions throughout the axial and appendicular skeleton, including the skull base, as well as fluorodeoxyglucose-avid lymph nodes above and below the diaphragm before chemotherapy and **d** marked response of all hypermetabolic lesions after chemotherapy

**Fig. 2:**
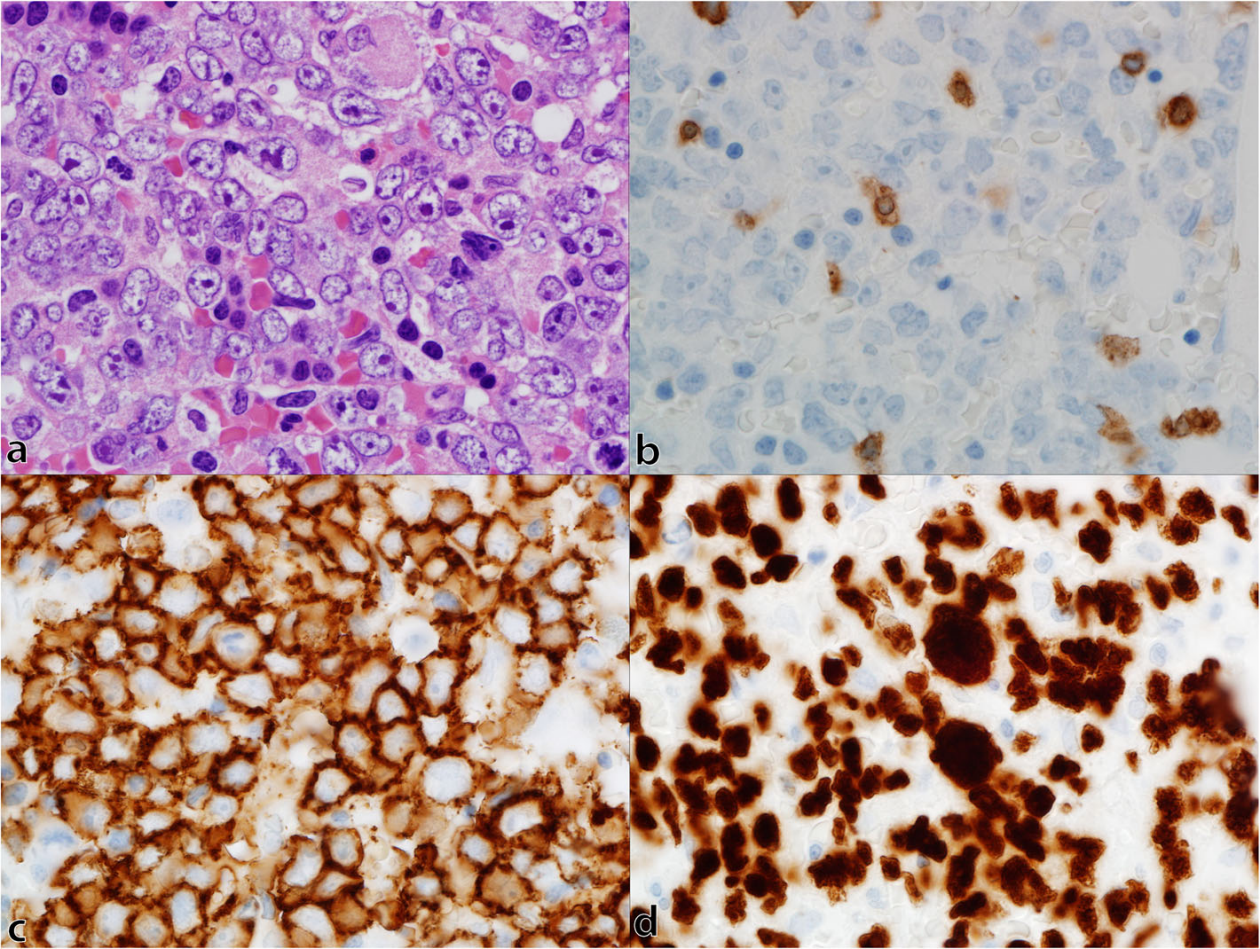
Bone marrow involvement by diffuse large B cell lymphoma. Photomicrographs of bone marrow trephine biopsy show: **a** medium to large neoplastic lymphocytes with nuclear irregularity, dispersed chromatin and variably sized nucleoli and background hematopoietic precursors on hematoxylin and eosin stain, **b** rare scattered T cells highlighted by CD3 immunohistochemistry, **c** membranous staining of neoplastic B cells by CD20 immunohistochemistry, and **d** nuclear staining of neoplastic B cells by PAX5 immunohistochemistry. a–d; × 1000

Given the persistent headache despite high-dose glucocorticoids and atypical features, alternative etiologies were suspected. The local MRI of his brain was reviewed and evidence of abnormal enhancing soft tissue involving Meckel’s cave bilaterally with extension through the foramen ovale was noted (Fig. 1a). The differential diagnosis for infiltrative process in Meckel’s cave included sarcoidosis as well as primary or secondary neoplastic lesions such as meningioma, nasopharyngeal carcinoma, schwannoma, neurofibroma, and lymphoma [4, 5]. The presence of constitutional symptoms and elevated inflammatory markers suggested a secondary process with associated intracranial involvement. As such, positron emission tomography (PET)-CT was obtained. This demonstrated extensive hypermetabolic lesions throughout the axial and appendicular skeleton, including the skull base, as well as fluorodeoxyglucose (FDG)-avid lymph nodes above and below the diaphragm (Fig. 1c). MRI of his left hip revealed diffusely abnormal marrow signal suggestive of infiltrative disease. A cervical lymph node biopsy demonstrated evidence of diffuse large B cell lymphoma and staging bone marrow biopsy revealed a hypercellular marrow (90%) with diffuse large B cell lymphoma involving 70% of the total cellularity (Fig. 2). Initiation of high-dose methotrexate (3.5 g/m^2^), rituximab (375 mg/m^2^), cyclophosphamide (750 mg/m^2^), hydroxydaunorubicin (50 mg/m^2^), Oncovin (vincristine; 2 mg), and prednisone (255 mg) (MR-CHOP; 21-day cycle) resulted in interval improvement in his headache and visual symptoms (Fig. 1b, d).

Ten months after initial diagnosis and 4 months after completion of his sixth cycle of MR-CHOP, our patient re-presented with bilateral lower extremity weakness and PET-CT showed recurrence of disease with evidence of neurolymphomatosis of multiple cervical, thoracic, and sacral nerve roots. Intrathecal cytarabine (50 mg) was given and ibrutinib (140 mg/day) was initiated, resulting in initial findings of improved strength and subsequent decreased nerve root FDG-avidity.

## Discussion

This case highlights a notably rare manifestation of metastatic lymphoma presenting with involvement of Meckel’s cave, which was initially mischaracterized as biopsy-negative GCA. Awareness of the neurologic features associated with skull base tumors involving Meckel’s cave as well as identification of atypical features of elderly-onset headache that should raise suspicion for etiologies other than GCA are needed to educate providers in order to appropriately identify, diagnose, and manage these uncommon presentations that are associated with high morbidity.

Meckel’s cave, also known as cavum trigeminale, is a cerebrospinal fluid (CSF)-containing dural pouch in the middle cranial fossa connecting the cavernous sinus to the prepontine cistern of the posterior fossa [5]. This structure contains the Gasserian ganglion of the trigeminal nerve as well as the three postganglionic trigeminal roots: ophthalmic (V1), maxillary (V2), and mandibular (V3) [5]. Tumors in Meckel’s cave are uncommon but the most frequent neoplasms at this site include trigeminal nerve sheath tumors, schwannomas, meningiomas, neurofibromas, nasopharyngeal carcinoma, and leptomeningeal metastases from solid organ malignancy (renal cell, lung, and breast) [4–7]. Metastatic lymphoma involving Meckel’s cave is rare with as few as 11 previously reported cases [8]. The most notable features of Meckel’s cave involvement include trigeminal neuralgia, facial numbness, and diplopia [6, 9]; the latter typically due to sixth nerve palsy resulting from the close proximity of the V1 branch in the cavernous sinus.

MRI is the imaging modality of choice for evaluation of patients with trigeminal neuralgia in which concern of Meckel’s cave involvement is suspected and should be performed with T1-weighted and T2-weighted imaging, short-tau inversion recovery, and gadolinium-enhanced T1-images with fat suppression [5]. Radiographic features suggestive of pathology of Meckel’s cave include nerve enlargement, enhancement, or trigeminal cistern CSF effacement [5]. Unfortunately if these findings are subtle, particularly if they are symmetrical, they can be overlooked, as occurred initially in the presented case.

Due to its rarity, limited information is present in regards to the best treatment for patients with metastatic lymphoma involving Meckel’s cave. Treatment with high-dose methotrexate and whole brain radiation has been used with variable outcomes [10]. Consolidation therapy with rituximab, cyclophosphamide, hydroxydaunorubicin, Oncovin (vincristine), and prednisone (R-CHOP) has shown benefit in case reports [11] and led to improvement of headache and visual symptoms in our patient when combined with high-dose methotrexate. Relapsing/refractory patients, as observed in our case, may require additional therapies such as single agent ibrutinib, rituximab plus ifosfamide-carboplatin-etoposide (RICE), or chimeric antigen receptor T cell (CAR-T) therapy [12].

In addition to its rarity, the described case provides a cautionary tale in evaluating patients with headache and inflammatory marker elevation. While classification criteria for GCA include an age at disease onset of ≥ 50 years, new-onset headache, temporal artery abnormality including tenderness to palpation or decreased pulsation, ESR ≥ 50 mm/hour, and an abnormal TAB with characteristic features [13], the intended use of these criteria is to create homogenous cohorts of patients for research purposes by distinguishing individuals with GCA from other cases with known vasculitis. Currently there are no approved or endorsed diagnostic criteria for GCA. Consequently, classification criteria for GCA are often incorrectly used in routine clinical practice for assistance in diagnosis. Although the described patient met criteria for age, headache, and inflammatory markers, his additional atypical features, including negative temporal artery biopsies, lack of response to high-dose steroids, and presence of trigeminal neuralgia, should raise suspicion for alternative etiologies.

Positive TAB has historically been considered the gold standard for diagnosis of GCA. However, given temporal artery involvement is not uniformly contiguous (that is, skip lesions) it is possible for patients to have classical features of GCA but have a negative biopsy (so-called biopsy-negative GCA). Clinicians should be cautious to exclude other mimicking conditions in this circumstance, particularly if features are atypical. It is noteworthy that among patients with initial suspicion of GCA undergoing TAB, 80% may have an alternative diagnosis [14]. Among a study of 123 patients with a negative TAB who were not diagnosed with GCA, common alternative diagnoses included self-limited disease (23%), isolated polymyalgia rheumatica (18%), stroke or transient ischemic attack (17%), and, less commonly, infection (5%) or malignancy (2%) [14]. Furthermore, not all abnormal temporal artery biopsies are due to GCA. In fact, rare but important mimics to consider include perivascular and intravascular lymphoma which may present with vision loss, headache, and increased inflammatory markers; however, pathology shows a strong neoplastic lymphoid infiltrate [15–18].

Glucocorticoids have been the cornerstone of treatment in patients with GCA. With high-dose glucocorticoids, significant symptomatic improvement can occur within as few as 24–48 hours [19] but may require up to 2 weeks. As such, a presumed diagnosis of GCA should be questioned if a therapeutic trial of high-dose glucocorticoids fails to result in marked clinical improvement in that time frame [19].

**Fig. 3:**
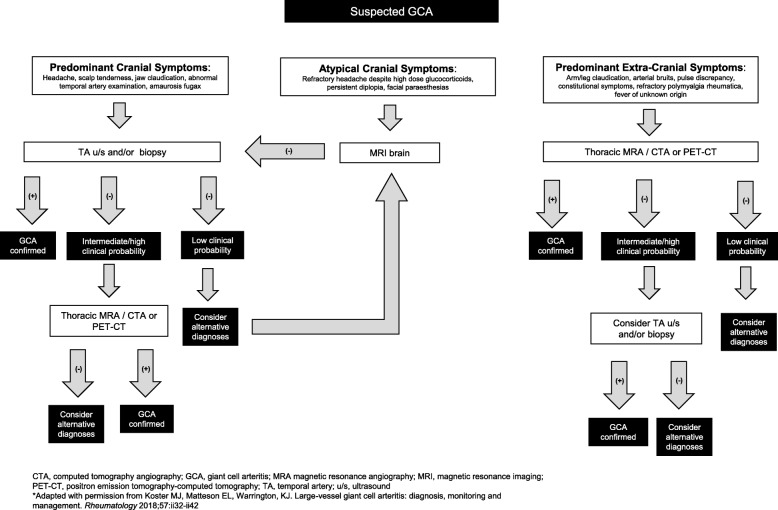
Algorithm for evaluation of suspected giant cell arteritis based on presentation features. *CTA* computed tomography angiography, *GCA* giant cell arteritis, *MRA* magnetic resonance angiography, *MRI* magnetic resonance imaging, *PET-CT* positron emission tomography-computed tomography, *TA* temporal artery, *u/s* ultrasound. (Adapted with permission from Koster MJ, Matteson EL, Warrington, KJ. Large-vessel giant cell arteritis: diagnosis, monitoring and management. *Rheumatology* 2018;57:ii32–ii42)

The European League Against Rheumatism (EULAR) recommends early imaging in patients with suspected GCA in capable centers to supplement clinical criteria [20]. For patients with cranial symptoms, evaluation of the temporal arteries by ultrasound or biopsy is prudent, but, if negative, additional imaging should be considered to support a suspected diagnosis of GCA. The use of PET-CT or thoracic magnetic resonance angiography (MRA)/CT angiography (CTA) are particularly helpful to evaluate suspected extracranial large vessel vasculitis but are also useful in identifying potential mimics, as was the circumstance in our case. The authors propose a suggested diagnostic algorithm to assist clinicians in evaluating patients with suspected GCA (Fig. 3).

## Conclusion

Our case illustrates an atypical presentation of diffuse large B cell lymphoma initially misdiagnosed as biopsy-negative GCA. Lack of improvement of inflammatory markers and persistent symptoms despite high-dose glucocorticoids should prompt a thorough investigation of alternative diagnoses. Early imaging in patients with suspected GCA should occur to assist in confirming the diagnosis of GCA and ruling out alternative etiologies. Finally, pathology within Meckel’s cave, though rare, can mimic symptoms of trigeminal neuralgia and lead to further diagnostic challenges in patients presenting with facial numbness.

## Data Availability

Not applicable.

## Abbreviations

BMI: Body mass index
CRP: C-reactive protein
CSF: Cerebrospinal fluid
CT: Computed tomography
CTA: Computed tomography angiography
ESR: Erythrocyte sedimentation rate
EULAR: European League Against Rheumatism
FDG: Fluorodeoxyglucose
GCA: Giant cell arteritis
INR: International normalized ratio
MRA: Magnetic resonance angiography
MR-CHOP: Methotrexate, rituximab, cyclophosphamide, hydroxydaunorubicin, Oncovin (vincristine), and prednisone
MRI: Magnetic resonance imaging
PET: Positron emission tomography
R-CHOP: Rituximab, cyclophosphamide, hydroxydaunorubicin, Oncovin (vincristine), and prednisone
TAB: Temporal artery biopsy
